# Development and characterization of a new set of genomic microsatellite markers in rice bean (*Vigna umbellata* (Thunb.) Ohwi and Ohashi) and their utilization in genetic diversity analysis of collections from North East India

**DOI:** 10.1371/journal.pone.0179801

**Published:** 2017-07-07

**Authors:** Banshanlang Iangrai, Arunava Pattanayak, D. Evanoreen Ann Khongwir, Gratify Pale, Emica Mary Gatphoh, Alpana Das, Nikhil Kumar Chrungoo

**Affiliations:** 1 Centre for Biotechnology, ICAR Research Complex for NEH Region, Umiam, Meghalaya, India; 2 Department of Botany, North Eastern Hill University, Shillong, Meghalaya, India; National Institute of Plant Genome Research, INDIA

## Abstract

Rice bean [*Vigna umbellate* (Thumb.) Ohwi and Ohashi] is an underutilized crop believed to be domesticated in the Myanmar-Thailand region of Asia. In India, rice bean is mainly cultivated in the North-Eastern Hills, which is a hotspot for biological diversity. A 5' anchored PCR was used to develop microsatellite markers in rice bean. Twenty-eight specific primer pairs were designed and employed to characterize sixty five ricebean accessions collected from North East India. A total of 179 alleles were amplified with an average of 6.393 alleles per locus. The gene diversity was high (mean 0.534) in the accessions collected from Darjeeling, Nagaland and Manipur, which are bordering areas with East Nepal and Myanmar, respectively. Exceptionally high outcrossing rate was observed in the entire population. Population structure analysis identified three distinct clusters in which accessions collected from areas bordering Myanmar and East Nepal grouped separately. Using a combination of STRUCTURE and Principal Coordinate Analysis, relative affinity of the intermediate accessions could be established. However, differences in allelic counts among populations were non-significant. The results showed that there is a high level of genetic diversity within the accessions, with high outcrossing rate.

## Introduction

Rice bean [*Vigna umbellate* (Thunb) Ohwi and Ohashi] is a less utilized grain legume cultivated mainly in Nepal, Bhutan, North East India up to Myanmar, Southern China, Northern Thailand, Laos, Vietnam, Indonesia and East Timor [1]. It is mainly grown under shifting cultivation and used as vegetable, pulse, fermented pulse, fodder and in folk medicine. It is one of the eight *Vigna* species domesticated in Asia, and is very closely related to adzuki bean [*V*. *angularis*(Willd.) Ohwi and Ohashi] [2,3,4]. The original center of domestication of the Asian *Vigna*species is thought to be the Indo China region, and they belong to the subgenus *Ceratotropis*, comprising 3 sections, *Ceratotropis*, *Aconitifoliae*and *Angulares*. *Angulares*is the most recent and diversified section of the subgenus [5], to which rice bean belongs. Rice bean is believed to be domesticated from a wild form *V*. *umbellate* var*gracilis*, which is a cross fertile type. These wild types have been reported to occur in natural and disturbed habitats and are of indeterminate, photoperiod-sensitive, freely-branching, twining plant types with small seeds. Most rice bean landraces cultivated in North Eastern India are similar to these wild types.

In India, as a cultigen its distribution is mainly confined to the tribal regions of North Eastern hills and hilly tracts of Western and Eastern Ghats [6]. In the North Eastern Hill Region of India (Arunachal Pradesh, Manipur, Meghalaya, Mizoram, Nagaland, Sikkim, and Tripura) rice bean is grown predominantly under rainfed conditions in a mixed farming system, under shifting cultivation or in kitchen gardens and backyards. It is grown on a limited scale in the eastern and western peninsular tracts of India.

In a study of the genetics of domestication of rice bean, Isemura et al. [7] reported high collinearity between adzuki bean and rice bean in the order of molecular markers. Similarity in the domestication genes between the genome of the two species was also reported in the same study. They concluded that the elite genes of rice bean hold great potential for its improvement as well as its allied species. However, improvement and utilization of rice bean through conventional or molecular breeding still lag behind other crops like cowpea and mung bean in the genus Vigna [8].Genetic diversity studies with a limited number of accessions from China, Nepal and Thailand have been reported earlier [9,10,11]. Wang et al. [8] studied genetic diversity in 230 accessions of rice bean collected from 12 provinces of China using mung bean SSRs. They reported a clustered yet generalized distribution of the accessions suggesting a wide exchange of germplasm across the provinces. However, the most comprehensive study so far with a very wide range of cultivated and wild rice bean accessions is by Tian et al. [1]. They studied 472 accessions (cultivated and wild) from 16 Asian countries and reported high gene diversity (>0.5) in the South and South East Asian accessions. In all these studies, number of Indian germplasm was negligible. Muthusamy et al. [12] studied genetic diversity in 10 rice bean germplasm collected from Meghalaya, a North Eastern Himalayan state of India and reported high genetic diversity. Studies of genetic diversity with a considerable number of germplasm from Indian Himalayan region, where rice bean is mainly cultivated, has not been conducted.

Simple sequence repeats (SSRs) or microsatellites are motifs of short tandem repeats that may vary in the repeat units at a given locus. SSRs have specific advantages over other markers as they occur frequently and randomly in all eukaryotic DNA [13], are multiallelic and are amenable to both manual scoring and automation [14]. SSRs have been used in diversity analysis [15,16], genetic fidelity analysis [17], gene mapping and species identification [18] and linkage analysis [19].

Among different crop species, the frequencies and occurrence of the two most common dinucleotide repeats {(AC)n and (GA)n} have been worked out in relatively greater detail [20]. Trinucleotide and tetranucleotide repeats are also found in plant genomes, the most frequent among them being (AAG)n, and (AAT)n [13]. In another survey of published DNA sequences in 54 plant species, Wang et al. [21] observed that the (AT)n, sequences were the most abundant followed by (A)n, (AG)n, (AAT)n, (AAC)n, (AGC)n, (AAG)n, (AATT)n, (AAAT)n and (AC)n.

Many different strategies for obtaining microsatellite DNA loci have been described. The simplest approach; cloning small genomic fragments and using radiolabeled oligonucleotide probes of microsatellite repeats to identify clones with microsatellites, was the first described and work well in organisms with abundant microsatellite loci [22,23,24]. Unfortunately, this approach does not work properly when microsatellite repeats are less abundant. Later, two types of enrichment strategies were developed: 1) uracil-DNA selection [25] and 2) hybridization capture [26,27]. Hybridization capture is the predominant strategy in use because it allows selection prior to cloning, and therefore, is faster and easier to do with multiple samples than uracil-DNA selection, which requires passage of each library through two bacterial strains. With the availability of largescale sequence database, development of microsatellite markers from expressed sequence tags (EST) has now become routine in many laboratories as it is fast and does not require elaborate facilities. Introduction of high-throughput next-generation DNA sequencing made development of microsatellite markers much easier and faster. While the EST-based SSRs still suffer from the drawback that gene / genes not expressing at a particular organ / time are not included, high-throughput DNA sequencing requires substantial investment. The 5’-anchored polymerase chain reaction [28,29] is one of the simplest and fast methodsfor developing SSR enriched genomic libraries and subsequent isolation of microsatellites that does not require substantial investment.

## Materials and methods

### Plant materials and DNA extraction

**Fig 1:**
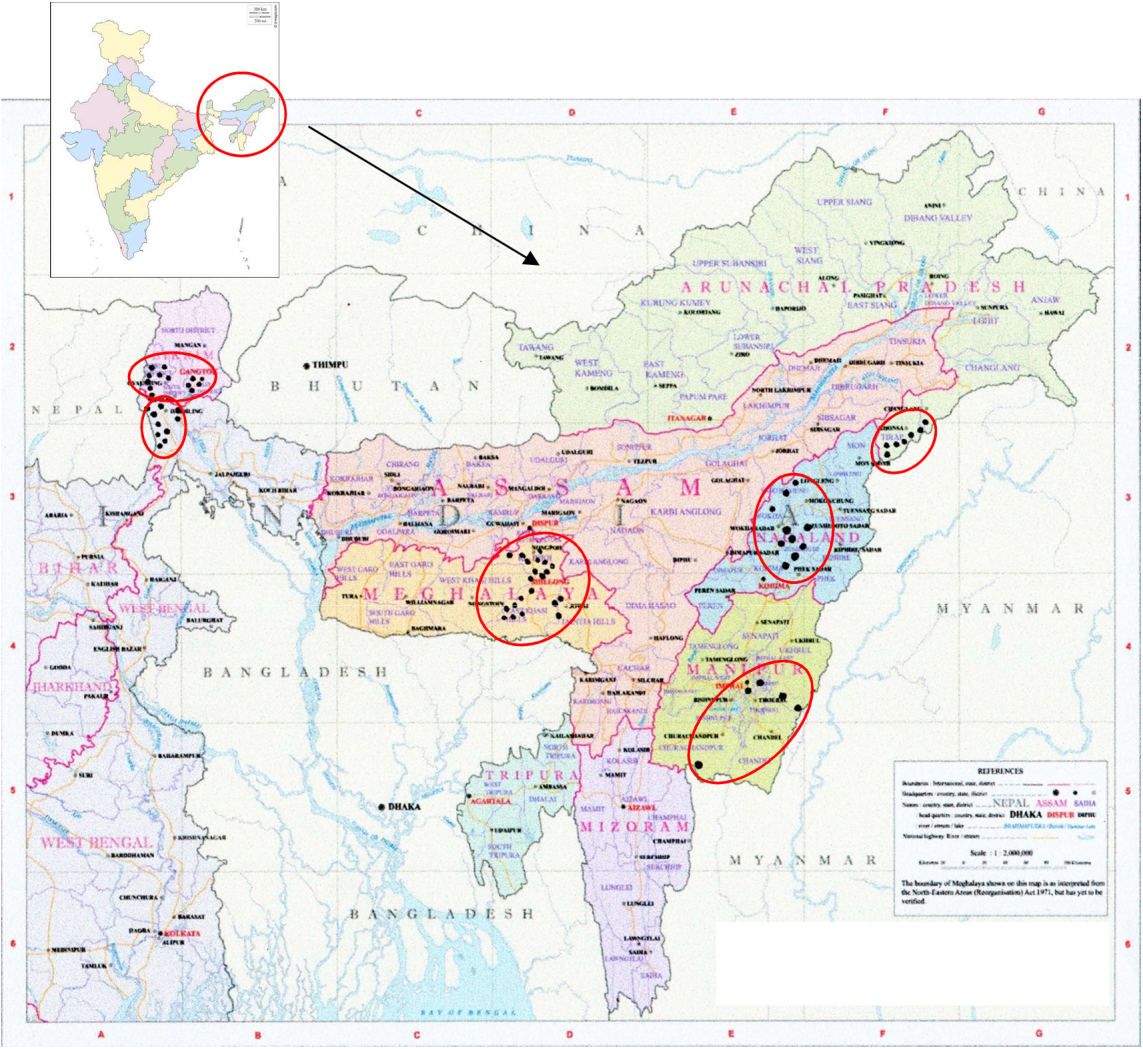
Geographic location of collection sites in the North Eastern Hills of India. The map used in the smaller box is reprinted from http://www.d-maps.com/carte.php?num_car=24867&lang=en. following their policy of free use (S1 File). Original copyright holder d-maps3.com. The map of North Eastern part of India (larger box) has been taken from the publication of National Atlas and Thematic Mapping Organization, Kolkata, India (original copyright holder). Circles in the both the maps indicate area of collection. Black dots inside circles indicate approximate location of collection.

Sixty-five accessions of rice bean collected from six North Eastern Himalayan states of India (Fig 1) were used for the study. All genotypes used for the diversity analysis are described in Table 1. These were maintained at the Division of Plant Breeding (re-named Crop Improvement), ICAR Research Complex for NEH Region, Umiam, Meghalaya. The total genomic DNA was extracted from leaves of 14-day old seedlings (one per accession) using the Plant DNA Isolation Kit (GCC Biotech, India) following the protocol of the manufacturer. DNA sample concentration was determined using a NanoDrop2000 (Thermo Fisher Corporation Inc.), and the DNA samples were diluted to 10 ng/μl prior to polymerase chain reaction (PCR) amplification.

**Table 1:** List of rice bean accessions used for genetic diversity analysis.

Sl No	Name	IC-No	Collection location
1	BKSB-255	IC350170	Lapnan, Tirap District, Arunachal Pradesh
2	BKSB-268	IC0137143	Meghalaya (maintained from original collection BD-15-A)
3	BKSB-221 (local name–Tohja)	IC350791	Jaintia Hills, Jaintia Hills District, Meghalaya
4	BKSB-202	IC351753	Rishi, West Sikkim District, Sikkim
5	Rhujon Lotha green	IC423278	Agunto village, Zoneheboto District, Nagaland
6	RBS-16	IC0141073	Meghalaya (maintained from BD-16-A)
7	BKSB-168	IC351543	Teesta, Darjeeling District, West Bengal
8	BKSB-225	IC350817	Mynso village, Jaintia Hills District, Meghalaya
9	BKSB-48	IC298009	Peducha, Kohima District, Nagaland
10	BKSB-156	IC352953	Hawaivingtekpi, Imphal District, Manipur
11	RHIDI(N)-2	IC423291	Tizu, Zoneheboto District, Nagaland
12	BKSB-194 (Local name–Metho bean)	IC351696	West Sikkim District, Sikkim
13	BKSB-164 (Local name–Seti bean)	IC351514	Algarh, Darjeeling District, West Bengal
14	BKSB-192	IC351671	Teesta Bazar, Darjeeling District, West Bengal
15	BKSB-245	IC350124	Phanyal, Changlang District, Arunachal Pradesh
16	BKSB-214	IC350763	Umden Mission, Ri-Bhoi District, Meghalaya
17	BKSB-195	IC351702	Legship, East Sikkim District, Sikkim
18	BKSB-246	IC350125	Changlang, Changlang District, Arunachal Pradesh
19	BKSB-188 (local name–Moissaeum)	IC351659	South Sikkim District, Sikkim
20	BKSB-233	IC350011	Miao, Changlang District, Arunachal Pradesh
21	BKSB-204 (Local name–Latera)	IC351761	West Sikkim District, Sikkim
22	BKSB-201 (Local name–Kalimani)	IC351748	West Sikkim District, Sikkim
23	BKSB-163 (Local name–Rato)	IC351512	Darjeeling District, West Bengal
24	BKSB-160 (Local name–Gala Dal)	IC351508	Darjeeling, Darjeeling District, West Bengal
25	BKSB-158 (Local name–Ransang)	IC351502	Darjeeling District, West Bengal
26	RHIDI KEMAGH	IC423239	Zoehnoboto, Zoehnoboto District, Nagaland
27	BKSB-70 (Local name–Bethe)	IC298121	Sarchi, Manipur
28	BKSB-207 (Local name–Moisseum)	IC351773	West Sikkim District, Sikkim
29	BKSB-46 (Kerhu– 2, black rice bean)	IC298097	Kohima, Kohima District, Nagaland
30	BKSB-45 (Kerhu– 1, white rice bean)	IC298096	Kohima, Kohima District, Nagaland
31	Jaluki (N)	IC423321	Yisemyong, Mokokchung District, Nagaland
32	Chungata	IC423323	Mongsinyini, Mokokchung District, Nagaland
33	BKSB-145	IC352930	Kakching, Thoubal District, Manipur
34	RHIDI KEKVA(N)	IC423332	Mopongchukak, Mokokchung District, Nagaland
35	BKSB-184 (Local name–Bhatamash)	IC351599	East Sikkim District, Sikkim
36	BKSB-220	IC350788	Phlongingkhw, Jaiantia Hills District, Meghalaya
37	BKSB-223 (Local name–Toh)	IC350791	Jaiantia Hills District, Meghalaya
38	BKSB-182	IC351588	Sukna, Darjeeling District, West Bengal
39	BKSB-170 (Local name—Rato bean)	IC351545	Darjeeling District, West Bengal
40	BKSB-166 (Local name–Thuli bhatamash)	IC351534	Darjeeling District, West Bengal
41	BKSB-254 (Local name–Pinchari)	IC350166	Changlang, Changlang District, Arunachal Pradesh
42	BKSB-36 RK-36, Local name–Rajamoong)	IC0129076	Shillong, East Khasi Hills District, Meghalaya
43	BKSB-165 (Local name–Ketimausem)	IC351529	Darjeeling District, West Bengal
44	BKSB-40 (Local name–Rumbaija)	IC298091	East Khasi Hills District, Meghalaya
45	BK-07-05 (Local name–Mashiam dal)	IC557283	East Sikkim District, Sikkim
46	BKSB-102	IC298153	Sumer, Ri-bhoi District, Meghalaya
47	RBS-24 (BKS, Local name–Rumbaija)	IC0129056	Ri-Bhoi District, Meghalaya
48	MRB-4 (Elite line PISRB1)	IC0557725	Meghalaya
49	MRB-6 (Elite line PISRB6)	IC0557728	Meghalaya
50	BK-07-07 (Local name–Mashiam dal)	IC557285	East Sikkim District, Sikkim
51	BK-07-20 (Local name–Mashiam dal)	IC557298	West Sikkim District, Sikkim
52	RCRB-06-10 (white creamy ricebean)	Selection from IC0015510	Meghalaya
53	RCRB-13 (Elite line)	IC0112382	Meghalaya
54	RHIDI(N)-1	IC423364	Yimyu, Mokokchung, Nagaland
55	RBS-15 (Elite line BD-14-A)	IC0015533	Meghalaya
56	RCRB-16 (Elite line)	IC0112381	Meghalaya
57	RCRB-1-6 (Yellow creamy rice bean)	Selection from IC0129056	Meghalaya
58	MRB-9 (Elite line BD-15-A)	IC0137140	Meghalaya
59	BKSB-122 (Local name–Chakwiachouba)	IC352853	Imphal East, Manipur
60	MNPL-3	No pedigree information available	Entered in the All India Coordinated Trial in 2003 as an entry from Manipur
61	BKSB-231 (Local name–Sarai)	IC350008	Changlang District, Arunachal Pradesh
62	MRB-2 (Elite line PISRB2)	IC0137141	Meghalaya
63	BKSB-200	IC351741	West Sikkim District, Sikkim
64	RBS-2 (Elite line BD-21-A)	IC0144686	Meghalaya
65	BKSB-235 (Local name–Sarai)	IC350031	Changlang District, Arunachal Pradesh

### Microsatellite library construction by 5' anchored PCR

A microsatellite library was constructed using the method of Fisher et al. [28]. Briefly, degenerate primers designed to anchor at the 5' end of microsatellite sequences were used to amplify genomic DNA. Di-repeated primers (PCT2 and PCT4) containing 7 degenerate bases at the 5' end of repeats were comprised of i) PCT2 - [5’-KKVRVRV(CT)_15_−3’] and ii) PCT4 - [5’- KKRVRVR(CT)6−3’] (where K = G/T,V = G/C/A and R = G/A) were used in PCR amplification to isolate a range of repeat motifs. PCR amplification was carried out in a total volume of 20 μl containing 50 ng of template DNA, 2.5 mM MgCl_2_, 1 X PCR buffer containing 10 mM Tris-HCl (pH = 8.0), 50 mM KCl, 1 unit Taq DNA Polymerase (Prime Taq, GCC Biotech, India) 10 pmol of degenerate primer (PCT2/PCT4) and 0.2 mM of dATP, dGTP, dCTP and dTTP. Amplification was carried out in a thermocycler (Genamp PCR 9700, Applied Biosystem), with an initial denaturation of 94^°^C for 3 minutes followed by 35 cycles of 94^°^C for 30 secs, 58.4^°^C (PCT2)/ 54.5^°^C (PCT4) for 40 secs, one minute at 72^°^C and a final extension step of 72^°^C for 5minutes. Successful amplification was confirmed by electrophoresing the PCR products on 0.8% agarose gel with a 1kb ladder (GCC Biotech, India) as size standard. The PCR products were afterwards purified using a PCR Purification Kit (GCC Biotech, India), cloned into pGEM®-T vector (Promega, Madison, USA) and a library was made. One hundred and twenty four random clones were then sequenced using Bigdye Terminator v3.1 Cycle Sequencing Ready Reaction kit (Thermo Fisher Corporation Inc.) on an ABI 310 genetic analyzer. Microsatellite containing sequences and natures of microsatellites were identified and analyzed using the software MISA [30] and specific primers were designed using Primer3 [31].

### Marker analysis

Primer modelling was possible for 53 primers pairs, which were then synthesized (GCC Biotech, India) and checked for amplification in a set of 12 accessions (2 from each population). Twenty-eight primer pairs that consistently gave unambiguous scorable single bands in all the 12 accessions were later resynthesized with fluorescent labeling (S1 Table) on the forward primer.

### Fluorescently-labeled microsatellite marker analysis

Individual PCR amplifications for each microsatellite were performed using the Genamp PCR 9700 thermocycler (Applied Biosystems.). The PCR amplification was carried out in a total volume of 10 μl containing 10 ng of template genomic DNA, 1.5 mM MgCl_2_, 1 X PCR buffer containing 10 mM Tris-HCl (pH = 8.0) and 50 mM KCl, 0.5 unit of Taq DNA Polymerase (GCC Biotech, India), two pmol of flourscent dye labeled forward primer, two pmol of unlabeled reverse primer and 0.1 mM of dATP, dGTP, dCTP and dTTP. The PCR profile was: 94^o^ C for 5 minutes followed by 35 cycles of 94^o^ C for one minute, 50.0^°^C- 60^°^C (depending on primer) for one minute, 72^o^ C for two minutes, and a final extension step of 72^°^C for 10minutes. The amplified PCR products were analyzed in an ABI Prism 310 genetic analyzer (Thermo Fisher Corporation Inc.) using GeneScan^TM^ -500 LIZ as size standard.

### Statistical analysis

Alleles were manually assigned to regular fluorescence peaks, and the sizes of the alleles were calibrated by the GeneMapper Software version 4.0 against GeneScan^TM^ -500 LIZ Size Standard. Autobin v 0.9 software (Franck Salin, INRA Pierroton-UMR BIOGECO) was used to analyze the raw data. For statistical analysis, the accessions were grouped into six population *viz*. P1 = Arunachal Pradesh; P2 = Manipur; P3 = Nagaland, P4 = Sikkim; P5 = Darjeeling; P6 = Meghalaya according to their geographic location (state of India except Darjeeling, which is a district) of collection. Allelic patterns, analysis of molecular variance (AMOVA), principal co-ordinate analysis (PCoA) and Nei [32] genetic distances were calculated using GenAlex software [33]. Population wise expected (*He*) and observed (*Ho*) heterozygosity, *f* (fixation index) values were calculated using gDA v1.0 software [34]. Out crossing rate was calculated from fixation index using the equation *t = (1-f)/(1+f)*[1]. The number of alleles/locus, major allele frequency, gene diversity and PIC values were calculated using Power marker version 3.25 software [35]. Genetic distance was calculated using C.S. Chord distances, and phylogenetic tree was constructed based on neighbor joining (NJ) method as in the Power Marker Software. The tree was viewed using the software Tree view v1.6.6 (http://taxonomy.zoology.gla.ac.uk/rod/treeview.html). Population structure was analyzed using the software STRUCTURE V.2.3.4 [36,37] as described by Roy et al. [15] except that initially 40 replicate runs were done for *K* = 1–12, and the second run was done at *K* = 3, *K* = 6 and *K* = 8 with 40 replications. Finally, at *K* = 3, the membership co-efficient from the run with the highest LnP(D) value (-3940.2) was used to assign the accessions to cluster 1–3 (SP1-3) based on their highest membership co-efficient for a cluster. Accessions with <0.6 membership co-efficient for all the clusters were grouped as admixtures (SP4). For the structure clusters (SP1-4), allelic pattern, genetic distance, AMOVA and PCoA were calculated using the GenAlEx V6.5 [33].For the geographic location-based and Structure-based populations, significance of difference of allelic count (FPTest) was tested using FPTestR software [38].

## Results and discussion

### Characterization of microsatellite loci

DNA sequencing of the clones indicated the presence of a total of 124 microsatellite repeats (Table 2). Di-nucleotide repeats were the most abundant (79%) followed by penta-nucleotide repeats (17.7%) and tri-nucleotide repeats (3.2%). CT (48.98%) and GA (46.9%) were the most abundant type. Repeat units in the dinucleotide varied from 9–25 (both in AG). However, 16 units was the most common (40 in CT and 38 in GA). Among the pentanucleotides, TAAAA motif was the most abundant (14 of 22). A total of 124 clones yielded fragments containing 49 dinucleotides, two trinucleotides and two pentanucleotides. Primer modelling was possible for 53 microsatellite loci out of which 28 microsatellite loci were found to give unambiguous scorable bands. These were selected for further analysis.

**Table 2:** Distribution of isolated microsatellite motifs in the identified microsatellites.

Motif	Repeat unit	No. of sequences
AG	9	02
AG	25	02
CT	10	06
CT	16	40
CT	17	02
GA	10	06
GA	16	38
GA	17	02
TTC	9	4
ATTTT	5	8
TAAAA	5	14

Details of the developed SSR primers with *Tm*, expected product size and dye labeling used for our study are given in the S1 Table. All the SSRs showed polymorphism and average alleles per locus (NA) was 6.39 (Table 3) within a range of 2 (VUCT 18) to 13 (VUCT 19). Gene diversity ranged from 0.03 (VUCT26) to 0.784 (VUAG09) with a mean of 0.532. Observed heterozygosity ranged from 0.062 (VUGA 25) to 1.00 (VUTG 21) with a mean of 0.528. Mean polymorphism information content was 0.488 with a range of 0.030 (VUCT26) to 0.756 (VUAG 09).

**Table 3:** Summary statistics of the characterization of twenty-eight SSR markers on a set of sixty-five accessions.

Marker	Average number of alleles/locus (NA)	Gene Diversity (He)	Observed Heterozyogosity (Ho)	Polymorphism information content (PIC)	Band size range (bp)
VUCT01	6.0	0.701	0.985	0.651	100–132
VUCT10	7.0	0.647	0.969	0.589	181–298
VUCT11	6.0	0.553	0.600	0.466	159–399
VUCT12	6.0	0.442	0.508	0.384	320–369
VUGA13	5.0	0.638	0.631	0.590	195–201
VUTTC14	6.0	0.240	0.138	0.234	126–171
VTAAT15	4.0	0.612	0.938	0.535	364–380
VUATTTT16	6.0	0.596	0.477	0.525	288–301
VUGA17	5.0	0.707	0.615	0.654	203–268
VUCT18	2.0	0.180	0.077	0.164	244–249
VUCT19	13.0	0.612	0.646	0.597	85–168
VUCT02	4.0	0.060	0.062	0.060	366–374
VUTG20	4.0	0.558	0.277	0.469	141–258
VUTG21	9.0	0.738	1.000	0.698	209–254
VUTG22	9.0	0.613	0.323	0.581	208–370
VUCT23	3.0	0.498	0.308	0.388	203–208
VUGA24	9.0	0.721	0.938	0.682	300–375
VUGA25	6.0	0.090	0.062	0.089	316–339
VUCT26	2.0	0.030	0.031	0.030	374–377
VUGA27	9.0	0.649	0.323	0.586	108–465
VUGA28	4.0	0.498	0.277	0.389	195–274
VUCT03	7.0	0.352	0.292	0.343	116–139
VUCT04	10.0	0.765	0.938	0.732	112–497
VUAG05	10.0	0.608	0.492	0.584	242–386
VUTAAAA06	8.0	0.759	0.831	0.732	244–368
VUAAT07	5.0	0.576	0.554	0.510	292–312
VUAG08	7.0	0.691	0.662	0.662	204–371
VUAG09	7.0	0.784	0.831	0.756	101–171
**Mean**	**6.393**	**0.532**	**0.528**	**0.489**	

Results of the characterization of the 6 geographic (location of collection) populations with the microsatellite markers are given in Table 4. Highest average number of allele/locus (4.750) was observed in the Meghalaya population while the lowest (2.929) was in the Manipur population. Gene diversity was highest (0.549) in the population from Darjeeling followed by populations from Nagaland and Manipur (0.544 and 0.543 respectively). It was the lowest in the Sikkim population (0.506). Observed heterozygosity (*Ho*) was highest in the Manipur population (0.571) and lowest in the Arunachal population (0.495).Mean out crossing was 95.88%.

**Table 4:** Summary statistics of the characterization of six geographic populations using twenty eight SSR markers.

Population	Samples size	Proportion of polymorphic locus	Mean no. of alleles per locus	Gene diversity	Observed heterozyogosity	Fixation Index	Outcrossing (%)
Arunachal	7.0	0.928	3.429	0.532	0.495	0.075	86.04
Manipur	5.0	0.893	2.929	0.543	0.571	-0.060	100.00
Nagaland	10.0	0.928	3.714	0.544	0.532	0.023	95.50
Sikkim	12.0	0.893	3.679	0.506	0.503	0.007	98.60
Darjeeling	10.0	0.857	4.036	0.549	0.536	0.025	95.12
Meghalaya	21.0	1.000	4.750	0.532	0.537	-0.009	100.00
**Mean**		**0.917**	**3.678**	**0.535**	**0.525**	**0.061**	**95.88**

**Fig 2:**
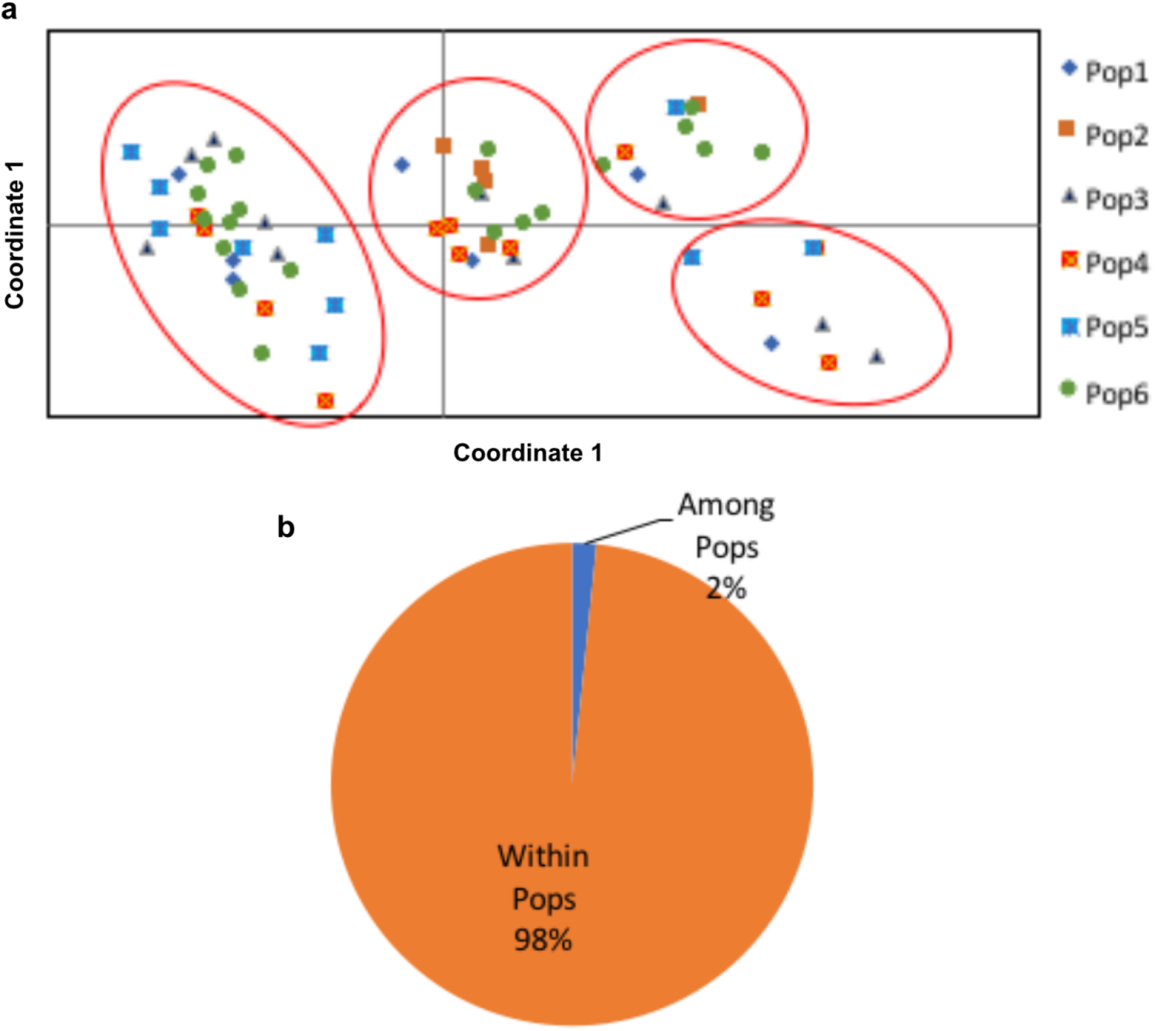
**(a)** Principal coordinate analysis of six geographic populations. Pop1 –Arunachal Pradesh, Pop2 –Manipur, Pop3 –Nagaland, Pop4 –Sikkim, Pop5 –Darjeeling, Pop6 –Meghalaya. **(b)** Analysis of molecular variance of six geographic populations.

Allelic patterns across the six populations are presented in Table 5. Number of alleles with a frequency of >5% were lower than the number of different alleles (alleles/locus) in the Sikkim (Pop5) and Meghalaya (Pop6) populations. Shannon’s Information Index (I) and alleles specific to a population (private allele) were the highest in the Meghalaya population (1.019 and 0.929 respectively, Table 5). Primers 17, 18, and 20 showed the highest number of private allele (4 each) while primer 1 and 5 showed the lowest number of private allele (1 each). Highest number of private allele was seen in the genotypes 28, 31, 60 and 64 (3 each).Principal coordinate Analysis (PCoA) showed that the 65 genotypes could be divided amongfour groups (Fig 2A). Manipur (Pop2) and Darjeeling (Pop5) populations showed clustering in two distinctly separated groups. First three axes of the coordinate described 28.32% of the variation. AMOVA showed that 98% of the variance was within population variance (Fig 2B).

**Table 5:** Mean values of allelic pattern of six geographic (collection location) populations.

	Pop1	Pop 2	Pop 3	Pop 4	Pop 5	Pop 6
NA	3.429	2.929	3.714	3.679	4.036	4.750
Na Freq/ > = 5%	3.429	2.929	3.714	2.714	4.036	2.893
Ne	2.389	2.332	2.470	2.303	2.802	2.442
I	0.897	0.845	0.947	0.887	1.009	1.019
No. of private alleles	0.143	0.107	0.357	0.071	0.286	0.643
No. of Less com alleles (< = 50%)	0.714	0.321	0.536	0.714	0.893	0.929

NA = No. of different alleles; Na Freq/> = 5% = No. of different alleles with a frequency of > = 5%; Ne = No. of effective alleles; I = Shanon’s information index.

Pop1 = Arunachal Pradesh; Pop2 = Manipur; Pop3 = Nagaland; Pop4 = Sikkim; Pop5 = Darjeeling; Pop6 = Meghalaya.

Analysis of the population using the model-based approach of the STRUCTURE software indicated the presence of structured population. Initially, the analysis was performed for evaluating 1 to 10 clusters (*K*) with five replicated runs per *K* value. ‘Structure Harvester’ program (http://taylor0.biology.ucla.edu/) was used to determine the optimum *K* value based on both LnP(D) and Evanno’s*ΔK*[39]. Two peaks were obtained, one at *K* = 3 (major peak) and the other at *K* = 5 (S1A Fig). Considering both the peaks, a second analysis was done with *K* = 2 to 6, length of burnin period 10,000, number of MCMC repeats 100,000, number of replicate runs 10 and keeping other conditions the same. Analysis with the Structure Harvester program produced a minor *ΔK* peak at *K* = 3 and a major peak at *K* = 5 (S1B Fig). To overcome this problem, the method suggested by Gante et al. [40] was used with slight modification. For the analysis, *K* = 1 to 12, burn in period 5000, MCMC repeats 50,000 and replicates 40 was were used. Structure harvester produced a clear *ΔK* peak at *K* = 3 with the lowest SD value for LnP (D). Two minor peakswere also seen at*K* = 6 and *K* = 8 (S2 Fig). Three further runs with *K* = 3, 6 and 8 were done keeping length of burnin period 10,000, MCMC repeats 100,000 and 40 replicates. Analysis of results in the runs with the highest LnP(D) values showed that at*K* = 3, nearly 50% of the accessions could be assigned to one of the three SPs considering a membership coefficient of ≥0.6 for a SP. However, at *K* = 6 and *K* = 8, keeping the same parameter, no accession could be assigned to several SPs. Therefore, the run with the highest LnP(D) value at *K* = 3 was used for assignment of accessions to SPs.

**Fig 3:**
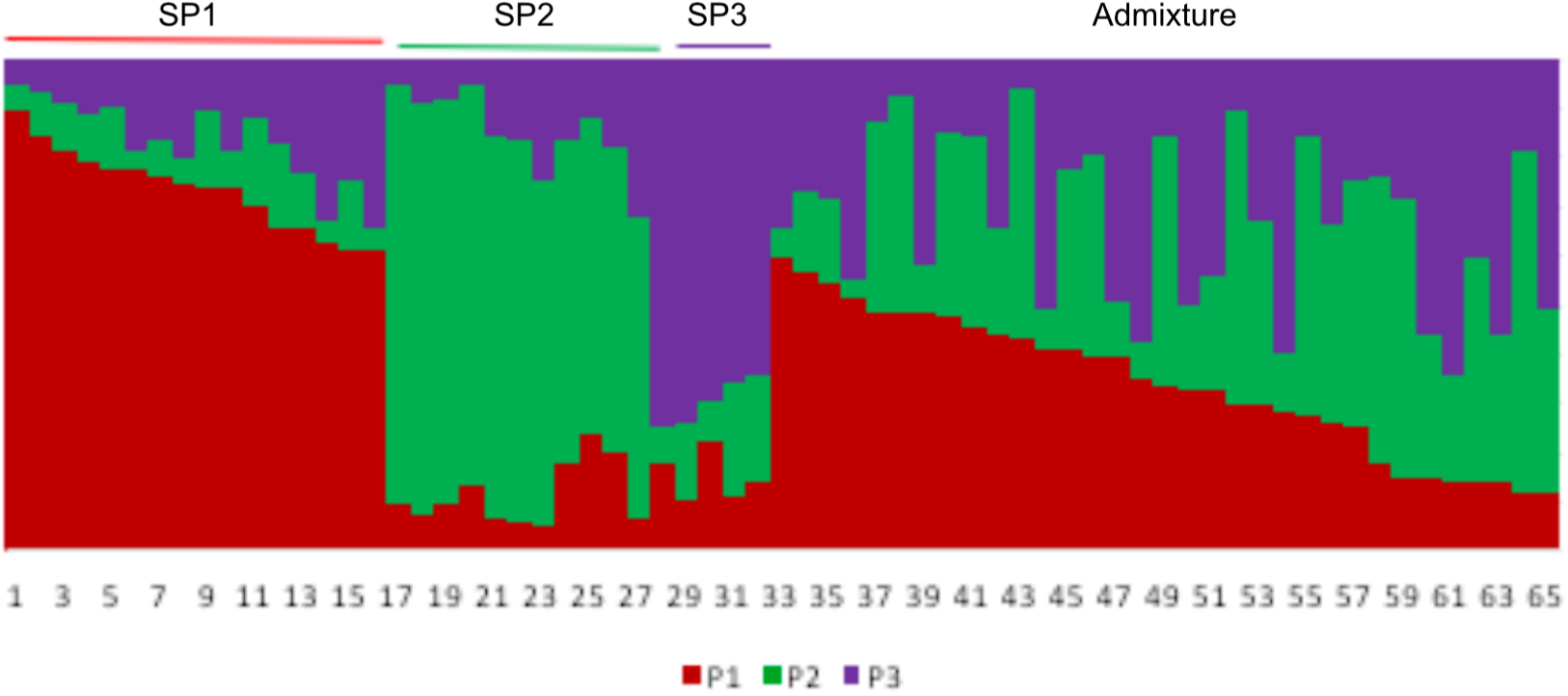
Model-based population structure of 65 ricebean accessions at *K* = 3. P1 –SP1, P2 –SP2, P3 –SP3. Each column represents an accession. Height of a particular colour in a column indicates proportion of contribution of each SP in an individual accession.

The LnP(D) and Evanno’s*Δ*K method supported the presence of 3 clusters (*K* = 3), represented here as SP1, SP2 and SP3, respectively. The inferred population structure is given in the Fig 3. SP1 contained 16 individuals (24.6% of the total accessions), dominated by accessions from Meghalaya (5) and followed byNagaland, Sikkim, Darjeeling (3 each) and Arunachal Pradesh (2). However, no accession from Manipur was included in this cluster. SP2 contained 11 individuals (16.9% of the total) with 3 accessions each from Meghalaya and Sikkim and 2 accessions from Darjeeling. One accession each from Arunachal Pradesh, Manipur and Nagalandwere also included in this cluster. SP3 was the smallest (5 accessions) representing 7.7% of the accessions studied. Three accessions from Meghalaya and 1 each from Manipur and Nagaland were included in this cluster.

The three clusters (SP1-SP3) had population specific *Fst* values (as calculated in STRUCTURE) of 0.400, 0.441 and 0.650, respectively with an average of 0.497indicating a low to moderate difference among clusters. Net nucleotide distance among SPs was highest between SP1 and SP2 (0.130) and lowest between SP1 and SP3 (0.052). At *K* = 3, 32 accessions were admixture (<0.6 membership co-efficient for any cluster).

**Fig 4:**
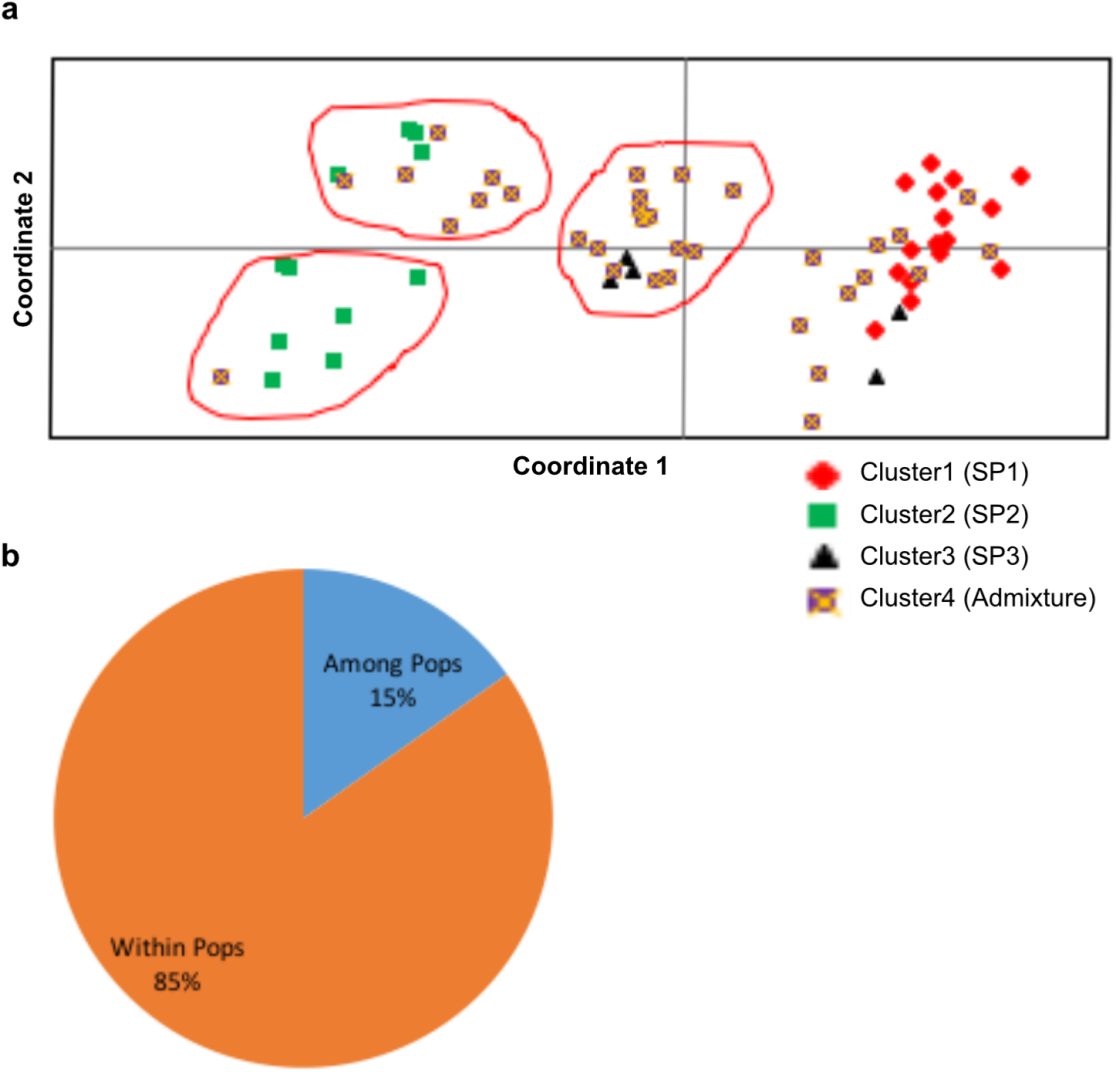
**(a)** Principal coordinate analysis of STRUCTURE clusters showing four prominent groups. Pop1 –Cluster 1 (SP1), Pop2 –Cluster 2 (SP2), Pop3 –Cluster 3 (SP3), Pop4 –Admixtures (SP4). **(b)** Analysis of molecular variance of STRUCTURE clusters (SPs).

**Fig 5:**
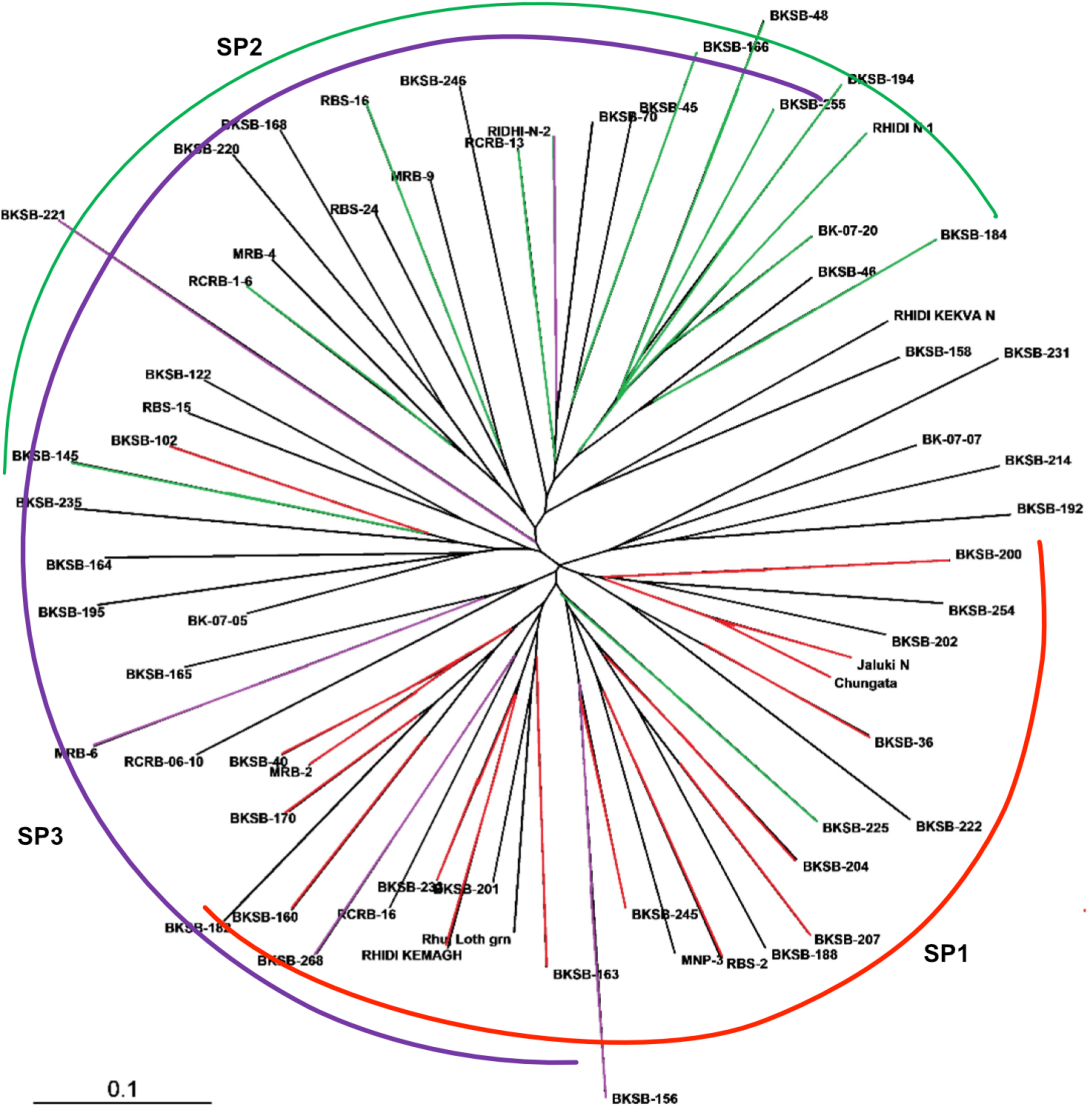
Neighbour joining tree showing genetic grouping of ricebean accessions. SPs are STRUCTURE clusters. Accessions with the same colour belong to the same SP.

Mean allele pattern across the SPs is given in Table 6. For this calculation, admixtures were treated as a separate population (SP4). Allele frequency was highest in SP1 (3.893). Shanon’s information index and number of private alleles were higher in the SP3. PCoA also supported the presence of three clusters. Four prominent groups were seen (Fig 4A). Accessions of SP1 and SP2 showed clear grouping while SP3 was spread across two groups. The first three axes explained 28.32% of the variation. AMOVA showed that 85% of the variance was due to within population variance (Fig 4B). Theneighbor joining (NJ)tree (Fig 5) also supported presence of three clusters. SP1 and SP2 showed clear grouping while two accessions of SP3 grouped alongwith SP1. FPTest [38] among different groups of geographic location-based populations or SPs indicated non-significant differences in allelic counts.

**Table 6:** Mean values of allelic pattern of STRUCTURE clusters (SPs).

Mean values	Clusters			
	SP1	SP2	SP3	SP4 (Admixtures)
NA	3.893	3.643	3.607	5.357
NA Freq/ > = 5%	2.643	2.571	3.607	3.250
Ne	2.197	2.240	2.761	2.563
I	0.807	0.846	1.034	1.067
No. of private alleles	0.179	0.071	0.429	1.036
No. of Less common alleles (< = 50%)	0.500	0.393	0.500	0.643
Gene diversity	0.416	0.445	0.556	0.536

NA = No. of different alleles; Na Freq/> = 5% = No. of different alleles with a frequency of > = 5%; Ne = No. of effective alleles; I = Shanon’s information index.

In the recent years, a lot of interest has been shown in the assessment of genetic diversity in rice bean and development of molecular markers for the purpose. AFLP [41], RAPD and ISSR [12] were used earlier for assessment of genetic diversity in rice bean. Tian et al.[1] used SSRs from azukibean and Wang et al. [8] used SSRs from mung bean for assessment of genetic diversity in rice bean. In the next year Chen et al. [9] reported the first set of micro-satellite markers isolated from genes of rice bean. The same group [10] later reported isolation of genomic microsatellite markers from SSR enriched library. The present study reports application of a simple and rapid method for constructing microsatellite enriched library and subsequent isolation of microsatellites. In the present study, dinucleotide repeats were the most abundant motif (79%) as was reported earlier (81.6%) by Wang et al. [10]. However, in the study with genic SSRs [9], dinucleotide repeats were much lower (26.8%). Thus, it appears that in the genomic SSRs dinucleotide is the most abundant class. Nevertheless, the repeat motif (CT/GA) was somewhat similar to the previous reports on genic SSRs (AG/CT) of rice bean [9] and mung bean [42]. In the genomic SSRs, AC/GT was reported to be the most frequent type [10]. The proportion of the pentanucleotide class (17.7%), as seen in this studyis higher than all previous studies (0.27 in genic and 0.5% in genomic SSRs respectively). Comparison of the results obtained by us and in the previous studies suggested that both class and motif of SSRs at inter and intragenic sequences are different, which may be due to their distinctive roles at these two levels.

Average numbers of alleles per locus amplified by the primers reported here are more compared to previously reported SSRs from rice bean (6.39 alleles compared to 3 in genic and a maximum of 4 alleles in genomic SSRs) and azuki bean [43]. However, this is much lower compared to the average 12.9 alleles reported by Tian et al. [1] while using azukibean SSRs. The reason for this probably lies in the fact that rice bean SSRs were tested on a set of germplasm collected from a specific area while the azukibean SSRs were tested on a global set, including wild types. PIC values of the present set of SSRs (0.488) is also lower than the azukibean SSRs (0.57) tested on the global set [1].

The set of 65 genotypes were grouped into six populations based on their geographic location of collection, and into two regions based on proximity of collection locations. The hills of Arunachal Pradesh, Manipur, Nagaland and Meghalaya are almost adjacent and, therefore, were grouped as a region. The hills of West Bengal (Darjeeling District) and Sikkim share a common border and the collections from there were grouped as another region. The mean gene diversity observed in the present set (0.534) is comparable to the gene diversity (0.535) observed in the cultivated accession of rice bean in a previous study [1]. However, the observed heterozygosity was higher (0.525 compared to 0.134). In a previous study with the accessions from the present set, Gupta et al. [44] reported rich diversity in ten morphological characters. At the individual population level, the set from Nagaland, Manipur (which are closer to Myanmar and had free movement in pre-independent India) showed gene diversity similar to the report of Tian et al.[1] for Myanmar. The gene diversity (0.549) seen in the population from Darjeeling (bordering with East Nepal) supports the finding of Bajracharya et al. [11] who reported high diversity in the collection from Nepal. High gene diversity was also observed in the population from Manipur and Nagaland.

### Nature of the accessions

Most of the rice bean cultivated in the North Eastern Himalayas are similar to wild types in morphological traits except for some from Manipur (*viz*. MNPL3 used in this study), which are bold seeded. However, rice bean is seldom maintained as a perineal crop. The *f* (fixation index) values obtained in the 6 geographic location-based populations indicate an exceptionally high out crossing rate, which needs further investigation. Furthermore, the low inter population variation and non-significant differences in allelic counts are probably because of the high outcrossing rate. It is interesting to note that the Manipur population, and the Darjeeling population mostly grouped separately in the PCoA. The Nagaland and Meghalaya populations (Pop3 and Pop6 respectively) showed a tendency to group in these two groups. Half of the Sikkim (bordering Darjeeling) population (Pop 4) also showed grouping with the Darjeeling population. Manipur is an Indian State bordering with Myanmar (Fig 1) while Darjeeling is a bordering district with East Nepal. In a previous study [1], Myanmar and Nepalese populations were reported to group separately. In the STRUCTURE grouping, almost half of the accessions from Arunachal Pradesh, Nagaland, Darjeeling and Sikkim were spread across SP1 and SP2 along with 50% of the accessions from Meghalaya. On the other hand, only one of the accession from Manipur was included in SP2. In SP3, although accessions from Manipur, Nagaland and Meghalaya were included, Arunachal, Sikkim and Darjeeling were unrepresented. It appears that the rice bean population of North Eastern Indian Himalaya is probably related with populations in Nepal and Myanmar as the collections from bordering areas grouped separately. To understand this better, another PCoA (S3 Fig) with the same data was conducted where the admixtures were further grouped into three clusters based on their highest membership coefficient (SP4 –for accessions showing highest membership coefficient for SP1 and so on). Results showed that majority of the admixtures grouped with SP1 and SP3 while a small part of it grouped with few accessions of SP2. As seen from the accession details, the pure SP2 is represented by accessions from Darjeeling, Sikkim (both bordering Nepal), Meghalaya and Nagaland. The accessions of SP2 that grouped with admixtures are 3 from Meghalaya and one from Manipur (bordering Myanmar). The part of the SP3 that grouped with admixtures were one each from Manipur, Meghalaya and Nagaland and the part that grouped with SP1 were all from Meghalaya. Seehalak et al. [41] suggested that rice bean was probably domesticated in Myanmar and Northern Thailand. Tian et al. [1] detected some unique alleles in Western Nepal and Indian Himalayan accessions, which were not detected in Thai, Myanmar or other wild accessions. They indicated a possibility that the Himalayan region may be a domestication center. Our results suggest that the accessions from North Eastern Indian hills (region between Myanmar and Eastern Nepal) are probably related to the populations of both Eastern Nepal and Myanmar. However, a further study with accessions from Nepal, Myanmar and North East India would confirm this.

In summary, the study demonstrated that 5’ anchored PCR-based enrichment method is a fast and reliable method for developing new SSR markers, and for a better understanding of the nature of the admixtures identified by STRUCTURE software, PCoA is a valuable statistical tool. This is also the first comprehensive genetic analysis of rice bean accessions from North East Indian Himalayan region, which is considered as the center of diversity of many crops. The results further showed that there is a high level of genetic diversity within the accessions with exceptionally high outcrossing rate.

## Supporting information

S1 Table Characteristics of the twenty eight microsatellite primers isolated from V. umbellata. Shown for each primer pairs are the forward and reverse sequences with the flourochromes (6-FAM, NED, VIC and PET), repeat motif, annealing temperature and expected product size when run individually. Genebank accession numbers of clones: JQ839228.1 GI: 383479073 to JQ839255.1 GI: 383479100.
(DOCX)

S1 Fig Delta K values of initial STRUCTURE analysis. **(a)**–Burnin period 5,000, MCMC repeats 50,000, number of replicates 10, clusters 1–10. **(b)**—Burnin period 10,000, MCMC repeats 100,000, number of replicates 10, clusters 2–6.
(TIFF)

S2 Fig Delta K and Ln prob figures of the STRUCTURE analysis with a burnin period 5,000, MCMC repeats 50,000, number of replicates 40, clusters 1–12. (TIFF)

S3 Fig Principal coordinate analysis of STRUCTURE clusters and admixtures. Pop1 –SP1, Pop2 –SP2, Pop–SP3, Pop4 –SP4 (highest membership coefficient for SP1), Pop5 –SP5 (highest membership coefficient for SP2), Pop5 –SP5 (highest membership coefficient for SP3).
(TIFF)

S1 File Map permission. (DOCX)
