# Supplementary material for: Development and characterization of a new set of genomic microsatellite markers in rice bean (Vigna umbellata (Thunb.) Ohwi and Ohashi) and their utilization in genetic diversity analysis of collections from North East India
Source: PLoS One. 2017 Jul 7;12(7):e0179801. doi: 10.1371/journal.pone.0179801 (PMC5501436; doi:10.1371/journal.pone.0179801)
Supplement: S1 Table — Shown for each primer pairs are the forward and reverse sequences with the flourochromes (6-FAM, NED, VIC and PET), repeat motif, annealing temperature and expected product size when run individually. Genebank accession numbers of clones: JQ839228.1 GI: 383479073 to JQ839255.1 GI: 383479100. (DOCX) [file pone.0179801.s001.docx]

S1 Table : Characteristics of the twenty eight microsatellite primers isolated from *V. umbellata.* Shown for each primer pairs are the forward and reverse sequences with the flourochromes (6-FAM, NED, VIC and PET), repeat motif, annealing temperature and expected product size when run individually. Genebank accession numbers of clones : JQ839228.1 GI : 383479073 to JQ839255.1 GI : 383479100.

| Primers | Forward Primer | Reverse Primer | Repeat motif | Dye labelled | Tm (°C) | Product Size (bp) |
| --- | --- | --- | --- | --- | --- | --- |
| VUCT01 | CCTCTTCTTACACGCTATTGGG | CTTGGTGACGTAAAACATTCCA | (CT)16 | 6- FAM | 59 | 130 |
| VUCT02 | CCGATCTATTCACAGGTCACAG | AGGTCATTAAAAGCGTCTCCAA | (CT)8 | NED | 60 | 371 |
| VUCT03 | CCCATCCCTTTCTTTCTTTCTT | GTGTTAGCAATGAGTGGTGGTG | (CT)7 | NED | 59 | 129 |
| VUCT04 | CAAGGAGTGTGTTGTTGGATGT | GGCAAAGATTGGAGGTAAGAGA | (CT)16 | NED | 59 | 318 |
| VUAG05 | GGGCTCTCTCTCTCTAATTTTGG | TCGAATCTCTCTCGAATCTCTTG | (AG)6 | VIC | 59 | 376 |
| VUTAAAA06 | GGGGTGACTCTCTCTCTCTC | TCATCGTATCGAACTCGTTTTG | (TAAAA)5 | VIC | 59.5 | 376 |
| VUAAT07 | AGCCTCTCTCTCTCTCTCTCCC | GTCAATACGCCACTCGTTATCA | (AAT)5 | VIC | 56 | 339 |
| VUAG08 | GGGCTCTCTCTCTCTAATTTTGG | TCGAATCTCTCTCGAATCTCTTG | (AG)9 | 6- FAM | 58 | 376 |
| VUAG09 | GGAAAAGAAAATAGGGGAAGGA | TACACCCAAAGAAGCATACCCT | (AG)9 | PET | 58 | 138 |
| VUCT10 | AGTTCTCTAACCCACCGGATTT | GGAAAGAAAATAGGGGAAGGAA | (CT)8 | 6- FAM | 59 | 246 |
| VUCT11 | CCGATCTATTCACAGGTCACAG | CATTGTCCGAGGAAGAAGAAGT | (CT)8 | VIC | 60 | 247 |
| VUCT12 | CGATCTATTCACAGGTCCCAGTA | AGGTCATTAAAAGCGTCTCCAA | (CT)8 | 6- FAM | 60 | 373 |
| VUGA13 | GGAAGGAAGGACGAAAAGGAT | ATACGCCAAGCTATTTGGGTG | (GA)10 | PET | 59 | 194 |
| VUTTC14 | CGCCTCTCTCTCTCTAATCCAA | CTTGGTGCTGAAAGAAATGGTT | (TTC)9 | PET | 60 | 172 |
| VUAAT15 | GGGGTGACTCTCTCTCTCTC | TCATCGTATCGAACTCGTTTTG | (AAT)5 | 6- FAM | 59 | 376 |
| VUATTTT16 | TCGTATCGAACTCGTTTTGAGA | TCCTTCCCCTATTTTCTTTTCC | (ATTTT)5 | VIC | 59.5 | 302 |
| VUGA17 | TCATCGTATCGAACTCGTTTTG | TCCGTCTGTCTCTCCTTTCTCT | (GA)8 | PET | 59 | 399 |
| VUCT18 | AGTTATGAGGTGGGCTTTTGTG | CTCAATTTCCATGTCCTGCAT | (CT)15 | 6- FAM | 59 | 369 |
| VUCT19 | TGTGTGTGTGTCTGTGTGAGAAA | CTTGGTGACGTAAAACATTCCA | (CT)15 | VIC | 59 | 175 |
| VUTG20 | CTCTCTACGCCAGGTTCATTTT | CTAGCCAAAGAGCCAGTGAAAC | (TG)6 | NED | 59 | 252 |
| VUTG21 | TTAAGCTCTCTACGCCAGGTTC | AGGCCACGTCAGTTCAAAATA | (TG)6 | PET | 59 | 196 |
| VUTG22 | CTAACTGCTTTTGTTCGTTCCC | ACCCACAACTGGGGATCTTAAT | (TG)6 | 6- FAM | 59 | 325 |
| VUCT23 | TTAAGCTCTCTACGCCAGGTTC | ATGTTATTGCAGGGCACGTC | (CT)18 | VIC | 58 | 207 |
| VUGA24 | AATTCTCCGGTTCAAGGAATG | GATCTCTCACATAGGGGACACG | (GA)15 | NED | 59 | 345 |
| VUGA25 | GGAGAGAATGAGAGAGCACGAT | AAGTTGGTATGTTGGTTGGGAG | (GA)15 | PET | 59 | 302 |
| VUCT26 | TCTCTTCTCAACCTCACCAACA | CAAGAATACCCCGTCAATAAGC | (CT)8 | 6- FAM | 59.5 | 377 |
| VUCA27 | CAGCGGTGACTTCAAAAGCAGA | GGGTGGGGTGGCGACTTT | (CA)7 | 6- FAM | 55 | 393 |
| VUGA28 | GGAGAGAATGAGAGAGCACGAT | ACCATGATTACGCCAAGCTATT | (GA)21 | NED | 59 | 202 |
