# Supplementary figures and images for: Development and characterization of a new set of genomic microsatellite markers in rice bean (Vigna umbellata (Thunb.) Ohwi and Ohashi) and their utilization in genetic diversity analysis of collections from North East India
Source: PLoS One. 2017 Jul 7;12(7):e0179801. doi: 10.1371/journal.pone.0179801 (PMC5501436; doi:10.1371/journal.pone.0179801)

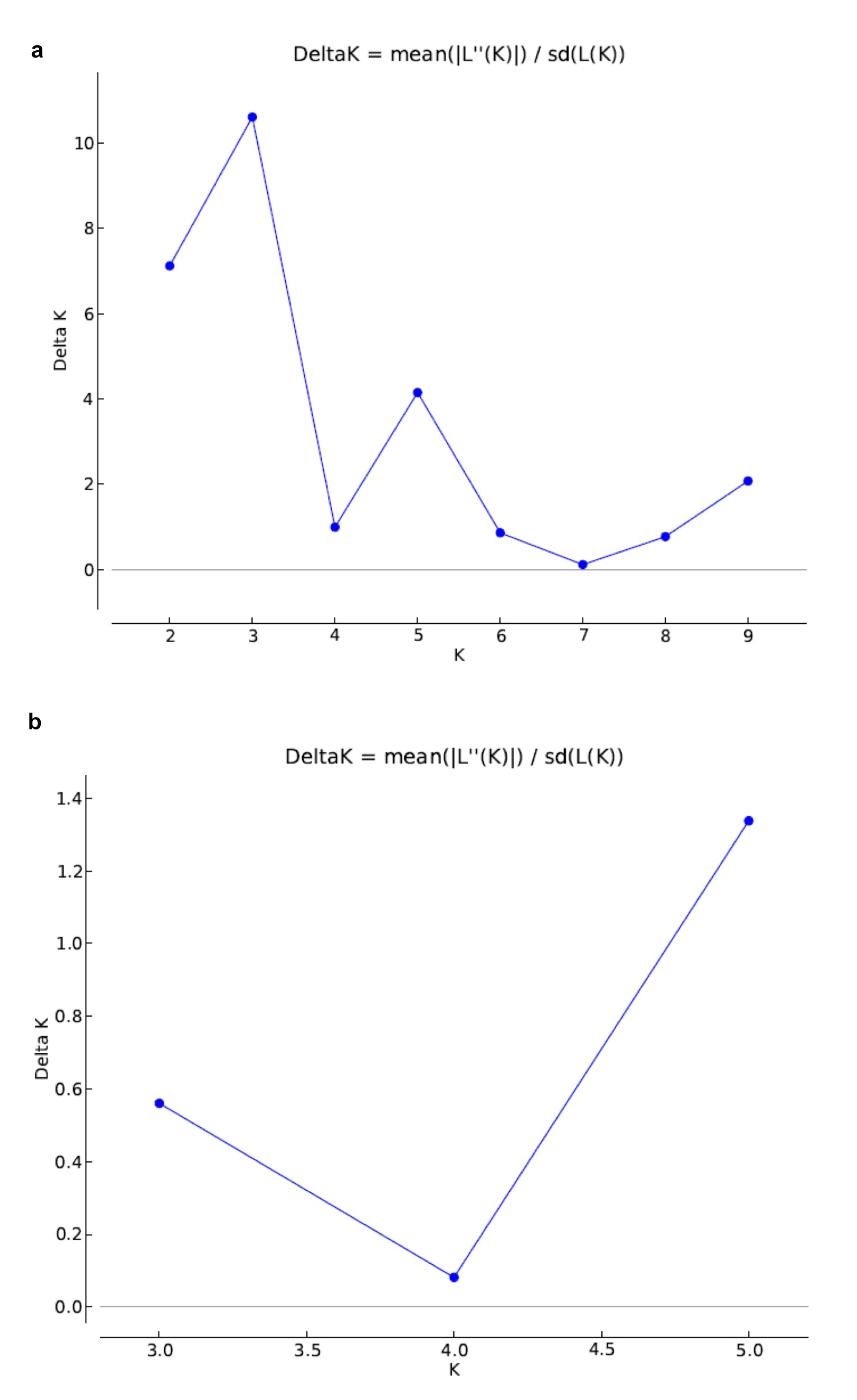

Supplement: S1 Fig — (a)–Burnin period 5,000, MCMC repeats 50,000, number of replicates 10, clusters 1–10. (b)—Burnin period 10,000, MCMC repeats 100,000, number of replicates 10, clusters 2–6. (TIFF) [file pone.0179801.s002.tiff]

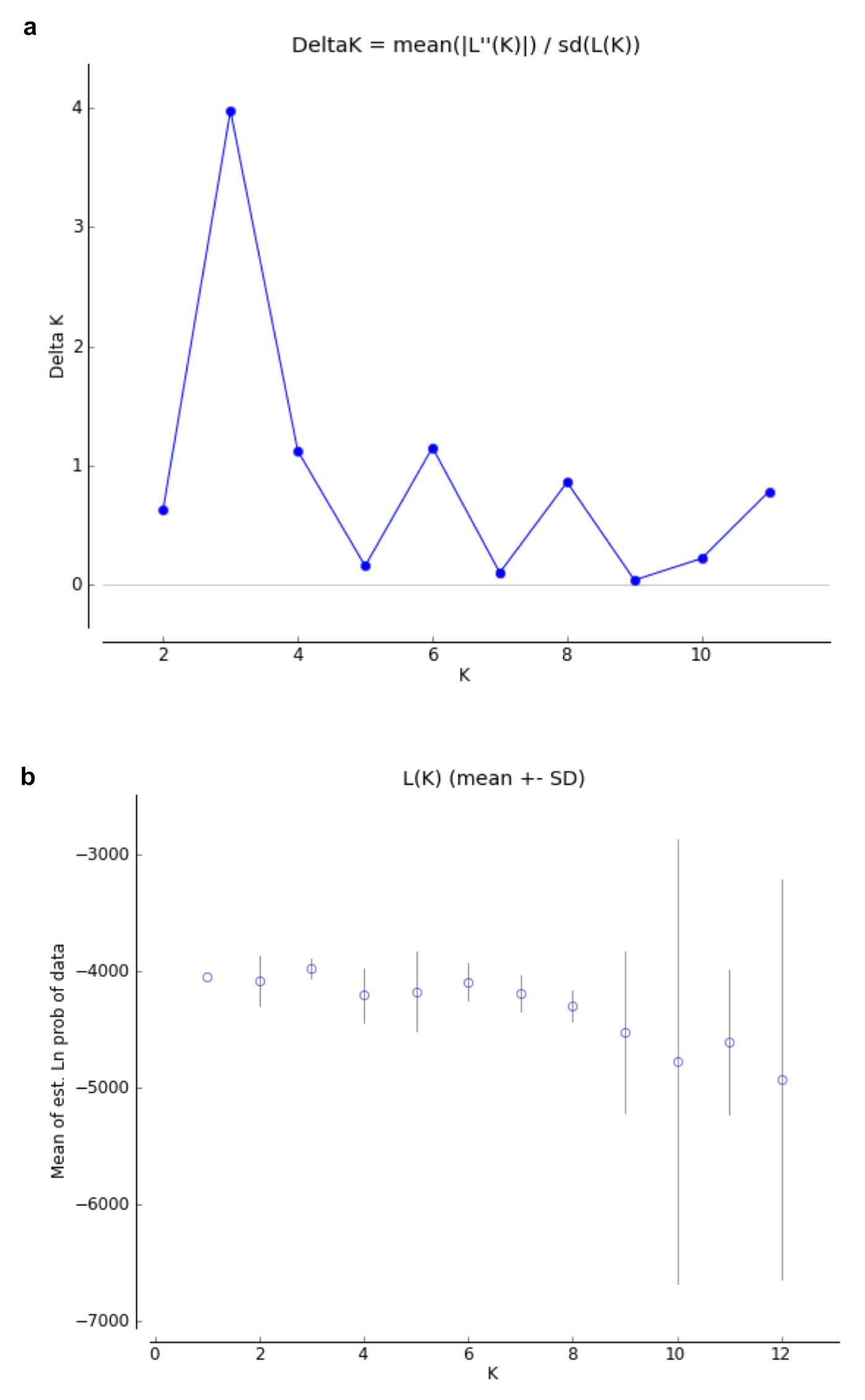

Supplement: S2 Fig — (TIFF) [file pone.0179801.s003.tiff]

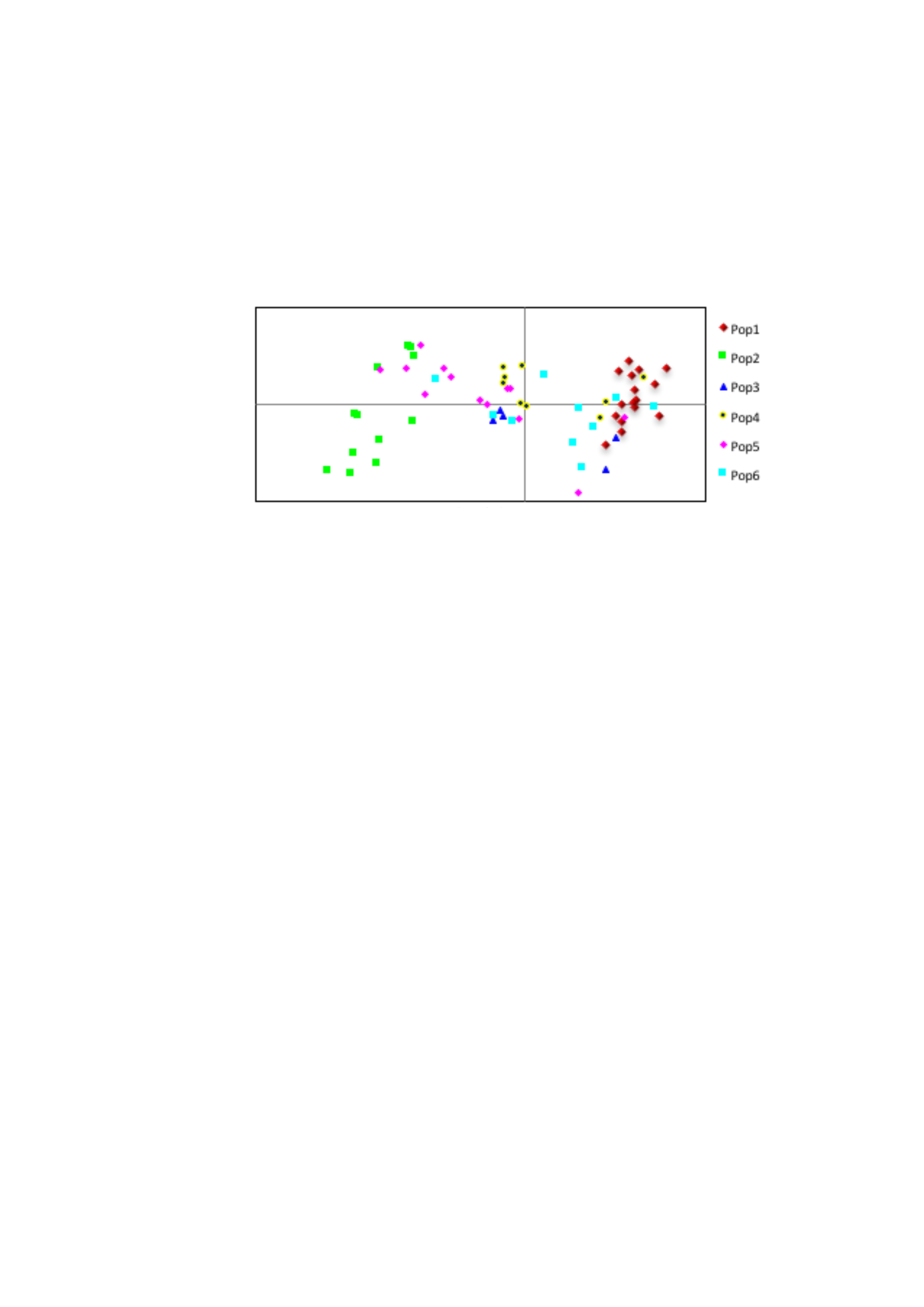

Supplement: S3 Fig — Pop1 –SP1, Pop2 –SP2, Pop–SP3, Pop4 –SP4 (highest membership coefficient for SP1), Pop5 –SP5 (highest membership coefficient for SP2), Pop5 –SP5 (highest membership coefficient for SP3). (TIFF) [file pone.0179801.s004.tiff]

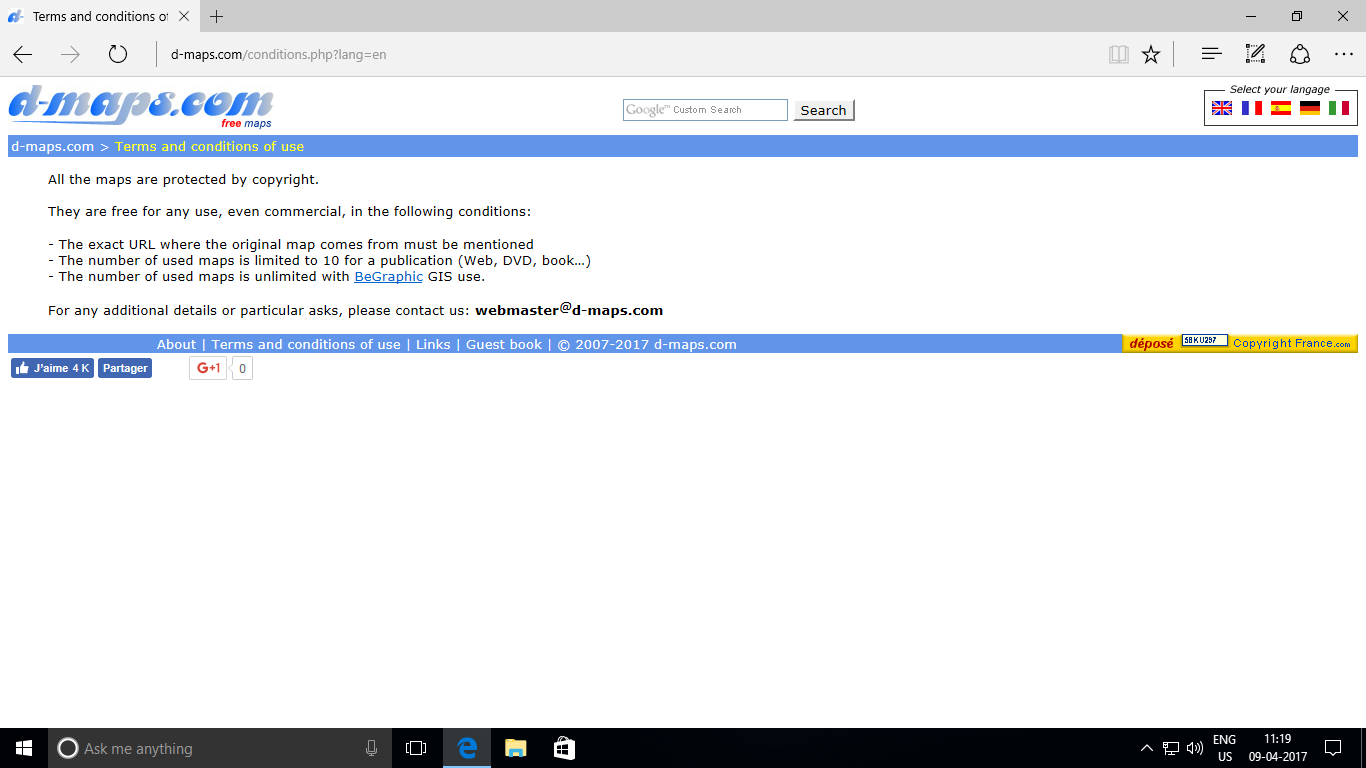
S1 File : Map permission.

Supplement: S1 File — (DOCX) [file pone.0179801.s005.docx]
